# Changes in the Phytochemical Profile and Antioxidant Properties of *Prunus persica* Fruits after the Application of a Commercial Biostimulant Based on Seaweed and Yeast Extract

**DOI:** 10.3390/ijms232415911

**Published:** 2022-12-14

**Authors:** Giuseppe Mannino, Maddalena Ricciardi, Noemi Gatti, Graziella Serio, Ivano Vigliante, Valeria Contartese, Carla Gentile, Cinzia M. Bertea

**Affiliations:** 1 Department of Life Sciences and Systems Biology, Innovation Centre, Plant Physiology Unit, University of Turin, 10135 Turin, Italy; 2 Department of Biological, Chemical and Pharmaceutical Sciences and Technologies (STEBICEF), University of Palermo, 90128 Palermo, Italy; 3 Green Has Italia S.p.A., 12043 Canale, Italy

**Keywords:** bioactive compounds, cellular antioxidant activity, radical scavenging activity, reducing power, polyphenols, liquid chromatography, mass spectrometry, Expando^®^, seaweed extracts, yeast extracts

## Abstract

Plant biostimulants are formulations that are experiencing great success from the perspective of sustainable agriculture. In this work, we evaluated the effect derived from the application of a biostimulant based on algae and yeast extracts (Expando^®^) on the agronomic yield and nutraceutical profile of two different cultivars (“Sugar Time” and “West Rose”) of *Prunus persica* (peach). Although, at the agronomic level, significant effects on production yields were not recorded, the biostimulant was able to reduce the ripening time, increase the fruit size, and make the number of harvestable fruits homogeneous. From a nutraceutical point of view, our determinations via spectrophotometric (UV/Vis) and chromatographic (HPLC-DAD-MS/MS) analysis showed that the biostimulant was able to boost the content of bioactive compounds in both the pulp (5.0 L/ha: +17%; 4.0 L/ha: +12%; 2.5 L/ha: +11%) and skin (4.0 L/ha: +38%; 2.5 L/ha: +15%). These changes seem to follow a dose-dependent effect, also producing attractive effects on the antioxidant properties of the fruits harvested from the treated trees. In conclusion, the biostimulant investigated in this work proved to be able to produce more marketable fruit in a shorter time, both from a pomological and a functional point of view.

## 1. Introduction

Over the past 15 years, the criteria for the consumer choice for food products has radically changed. Indeed, whereas food was exclusively considered as a supply of energy, macronutrients and micronutrients, now the role of plant food as a source of bioactive compounds is widely recognized [1]. In particular, recent scientific findings support the assumption that a diet based on the overconsumption of animal food may damage the physiological health status of consumers [2,3]. Meanwhile, phytochemicals contained in plant food have been positively related to a range of biological actions, including the regulation of cholesterol or glucose blood levels; antibacterial, antiviral or antiproliferative activity; UV/Vis protective effect; anti-inflammatory or antipyretic properties; and the prevention of the onset or development of chronic noncommunicable diseases [4,5,6,7]. As a result, plant food is increasingly recognized as a functional food. The consumption of fruit and vegetables represents the main way of intaking plant phytochemicals in the human diet. The importance of these bioactive components has motivated the World Health Organization (WHO) to recommend an intake of at least 400 g of fruit and vegetables in order to provide an adequate amount of bioactive molecules [8,9].

However, the quantity of phytochemicals contained in fruit and vegetables is strongly limited to the capacity of the plant secondary metabolism. Consequently, in order to enhance the bioactive compounds in plant food matrices, several strategies have been developed. For example, advances in plant biotechnology allowed the development of crop engineering techniques to shift plant biosynthetic pathways to the production of specific phytochemicals or simply to increase their production [10]. Although promising results have been achieved with genetically modified food (GMF), they are negatively perceived by both current European legislation and modern consumers. The main concerns include the potential risks to human health resulting from their consumption, ethical issues, lack of confidence in the regulatory machinery, resistance to change and lack of sustainability of the whole process [11]. As a result, several European countries today are still reluctant to cultivate and/or consume genetically modified organisms.

A secondary approach has been developed by the pharmaceutical and food industries to ensure the proper intake of phytochemicals. In particular, over the past few years a wide number of dietary supplements based on concentrated plant extracts have been introduced onto the market. The innovations in this field have guaranteed the supply of large amounts of bioactive compounds by the simple administration of a few tablets per day [5]. Nevertheless, consumers are questioning the real safety of such products, primarily due to the recognition that (i) the extraction and concentration processes utilize practices that are considered unsafe or dangerous to human health; (ii) there is an insufficient control system for these products; (iii) the concentration process leads not only to the increase in pharmaceutically relevant bioactive compounds but also other undesirable compounds, such as heavy metals, pesticides, insecticides, herbicides and aflatoxins [12,13,14].

A final strategy can be employed during agricultural stages by treating plants with specific products, such as fertilizers, agrochemicals or biostimulants, to ensure an adequate growth performance. These practices result in improved production, both from a quantitative and qualitative point of view [15]. However, the European Union is now increasingly attempting to discourage the excessive use of agrochemicals and fertilizers in agriculture, as they are considered harmful to the environment and human health [16,17,18,19].

In contrast to traditional agrochemicals, the newly born category of formulations has also found great success from a sustainability perspective, as they can be produced also using industrial waste [20,21,22,23,24]. These formulations, both of plant and animal origin, have been proven to be able to improve the resilience of plants to potential environmental stresses [25,26,27,28]. Among the several potential effects exerted by these formulations, recent scientific research has demonstrated that their application is capable of inducing a boost in the plant secondary metabolism resulting in an increase in bioactive compounds in different plant tissues, including flowers, leaves, roots, and fruits [29,30,31,32,33,34].

However, some limitations are currently present in this research area. For example, most scientific papers consider the biostimulatory effect (i) exclusively on annual plants, as they are experimentally easier to manage from an experimental point of view; (ii) in response to abiotic stresses; (iii) on a strictly agronomic and/or plant physiological level, without considering food quality; (iv) under strictly controlled conditions (e.g., greenhouses or phytotrons) [35]. Here, the biostimulatory effect of a commercial formulation based on seaweed and yeast extracts (Expando^®^) was evaluated on the phytochemical profile and antioxidant properties of fruits from two different varieties (“Sugar Time” and “West Rose”) of *Prunus persica* harvested from plants grown in the field.

*P. persica* fruits were chosen for this trial, as they (i) are fruits produced from perennial trees and not from annual crops; (ii) have a strong economic impact on the Mediterranean area, and Italy ranks first in Europe for their production [36]; (iii) are fruits ready for consumption after the harvest and do not need to ripen after picking [37]; (iv) the most suitable time for their picking is easily guessed by simple visual analysis and coincides with the change in skin color [37]. Moreover, according to the most up-to-date Faostat data, the world’s leading producer of peaches and nectarines, excluding China, is Italy, accounting for an average of nearly 1.5 million tons in recent years [38].

Regarding chemical analyses of peach fruits, they included the phytochemical profiling of both peach peel and pulp using UV/Vis methodologies and mass spectrometry (MS). In addition, antioxidant properties were evaluated by performing both solution and cell culture-based assays.

## 2. Results and Discussion

### 2.1. Biostimulant Application Contributes to Producing Homogeneous Yields and Improving Fruit Marketability

**Figure 1:**
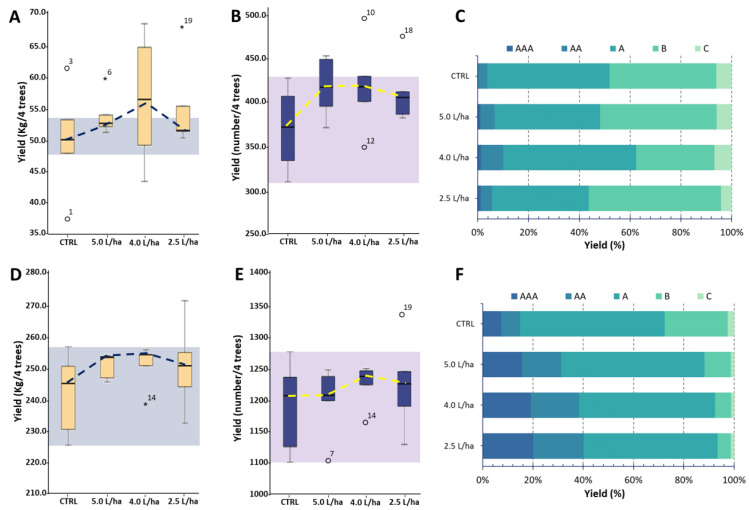
Agronomic yield data recorded for “Sugar Time” (**A**–**C**) and “West Rose” (**D**–**F**) varieties after treatment with different biostimulant dosages (5.0, 4.0 or 2.5 L/ha) or with water only. In the different panels, CTRL identifies samples harvested from trees treated with water only. (**A**,**D**) The production weight per four trees; (**B**,**E**) the number of produced fruits per four trees. Within each box, the horizontal, black lines indicate median values, while the boxes extend from the 25th to the 75th percentile of the distribution of the values in each group. Moreover, the extended vertical lines indicate standard deviations. The dotted lines represent the treatment trend, plotted considering the median value for each sample. Finally, the colored background reports the control values within which a significance of the data cannot be recorded. (**C**,**F**) The distribution of the fruit size (AAA, AA, A, B or C) in relation to the total production yield.

The production yield, maturity and quality of field-grown fruits can be affected by many environmental stressors. These stressor factors may result in both reduced agronomic yield and decreased fruit quality [39]. Scientific evidence has demonstrated how biostimulant formulations applied during fruit ripening can influence several plant physiological pathways, resulting in the improvement of a production yield [25,40]. The use of seaweed- and yeast-based biostimulants are gaining significant interest in agricultural systems due to the fact of their bioactive components that result in positive effects on crop production. These formulations have been shown to possess phytostimulant properties that are able to increase plant performances in several important crops [41]. However, the demonstrated effect seems to be influenced by the species of plant to which the formulation is applied [42]. Under our experimental conditions, the biostimulant treatments did not significantly (*p* < 0.05) affect the fruit yield of both varieties, either in terms of the number of harvestable fruits or the total weight (Figure 1).

However, although a higher amount of fruits was not recorded (Figure 1A,D), the plants treated with 4.0 or 5.0 L/ha were able to produce a more uniform number of harvestable fruits (Figure 1B,E). Regarding the fruit size, both peach varieties showed an augmentation in the most marketable fruit classes (AAA, AA and A) with a concomitant reduction in the smaller caliber classes (Figure 1C,F). Specifically, an increase of +187% and +118% was, respectively, observed for the AAA and AA fruit caliber classes of the “Sugar Time” variety (Figure 1C), while a rise of +137% and +123% was, respectively, observed for the AA and A fruit caliber classes of the “West Rose” variety (Figure 1F). These results are partially in agreement with those obtained in our previous work in 2021, in which we evaluated the effects of applying the same biostimulant on *Solanum lycopersicum* L. (var. “Micro-Tom”) fruits. Although the application protocol was the same, the two experimental designs varied in terms of the growing environment. Indeed, the tomatoes were grown with the irrigation, humidity, light exposure and temperature totally controlled by an automated system inside a greenhouse. In this case, an increase in the total production yield (+110%), a decrease in the fruit ripening time (approximately two weeks) and a significant increase in the fruit size (+85%) was observed [31]. On the other hand, these results appear to also be in agreement with Tarantino et al. (2018), who by estimating the effects derived from the application of three different commercial biostimulants on *Prunus armeniaca* trees (apricot) over two consecutive seasons, described an acceleration in the fruit ripening but a lack of an agricultural yield enhancement [43].

This result may suggest that Expando^®^ is able to influence fruit maturation by reducing the ripening time, thus achieving a more homogeneous production. This hypothesis is in agreement with Chouliaras and colleagues (2009), who tested a biostimulant with a similar composition on olive trees. The authors, following the application of the formulation, did not record significant effects on the total yield but observed an acceleration of the fruit ripening in accordance with an olive color change [44]. Similarly, the same authors evaluated the foliar application of the same biostimulant on kiwifruits, highlighting a remarkable effect on the fruit size and fruit maturation by 10–15 days [45]. Finally, other works using commercial seaweed extracts observed similar trends on clementine mandarins, Navelina oranges [46] and berry grapes [47,48,49].

### 2.2. UV/Vis Screening Assays Suggest an Increase in Phytochemicals in Peach Fruits

In order to understand whether the application of the biostimulant could affect not only the ripening but also the nutraceutical component of the fruits, spectrophotometric analyses aimed at the quantification of the total polyphenol content (TPC) were performed on the edible portions of the two peach varieties. Although it is generally discarded as a waste, the peel is also an edible tissue. Indeed, during the industrial production of juices, homogenized and nectars, the fruit is often used whole without being peeled. Even when discarded as a waste, it finds easy reuse within circular economy strategies, being a rich source of soluble and volatile bioactive compounds that are attractive to both the dietary supplement and cosmetic industries [50].

**Figure 2:**
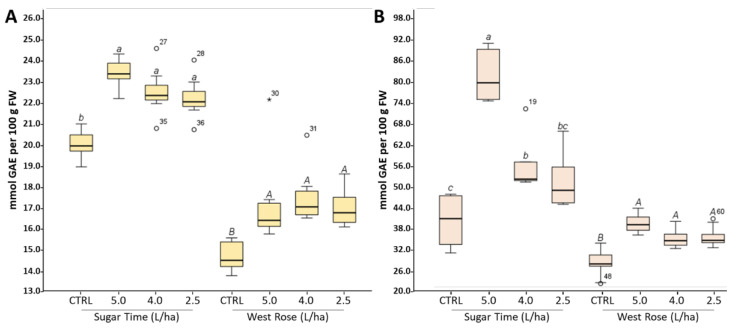
Total polyphenol content (TPC) measured in the pulp (**A**) or peel (**B**) of peaches harvested from trees treated with different dosages of biostimulant (5.0, 4.0 or 2.5 L/ha) or with water alone. In the different panels, CTRL identifies samples harvested from trees treated with water only. Values are expressed as mmol of gallic acid equivalent (GAE) per 100 g of fresh weight (FW). Within each box, the horizontal, black lines indicate median values, while the boxes extend from the 25th to the 75th percentile of the distribution of the values in each group. Moreover, the extended vertical lines indicate standard deviations. For each panel, within the same variety (“Sugar Time” or “West Rose”) the different lowercase (var. Sugar Time) or uppercase (var. West Rose) letters indicate significant differences at *p* ≤ 0.05, as measured by Tukey’s multiple interval test. The letter “a” indicates the highest value.

Under our experimental conditions, treatment with the biostimulant significantly (*p* < 0.05) affected the TPC in both the flesh and peel of the two varieties (Figure 2). Indeed, the TPC value measured in the pulp of treated peaches increased from 11% to 15% with respect to the CTRL, independently from the variety (Figure 2A). Similar results were also obtained for the peel in which the boost in the TPC of the biostimulant-treated peaches ranged from +23% to +153% (Figure 2B). Regarding the different dosages, our analyses suggest that for the “Sugar Time” peaches, the most effective treatment was 5.0 L/ha. Indeed, a significant (*p* < 0.05) change in the TPC was recorded in comparison with the lowest biostimulant dosages (Figure 2). Specifically, although in the pulp, the treatments with 4.0 and 2.5 L/ha were able to induce changes almost comparable to the 5.0 L/ha dosage (5.0 L/ha: +17%; 4.0 L/ha: +12%; 2.5 L/ha: +11%), in the peel an increase of +156% was observed using the highest dosage of the biostimulant, while minor increments were observed in fruits harvested from trees that were treated with the lower concentrations (4.0 L/ha: +38%; 2.5 L/ha: +15%) (Figure 2). Otherwise, although the biostimulant treatment led to an increase in TPC in both the edible tissues of the “West Rose” fruits, a dose-dependent effect was not observed in either pulp (Figure 2A) or peel (Figure 2B). Specifically, an increase of approximately 20% was reported in both tissues and for all tested dosages. These results are in agreement with previously published data demonstrating a positive role on the nutraceutical profile of fruits treated with biostimulants having a similar composition to that of our formulation [51,52,53].

**Figure 3:**
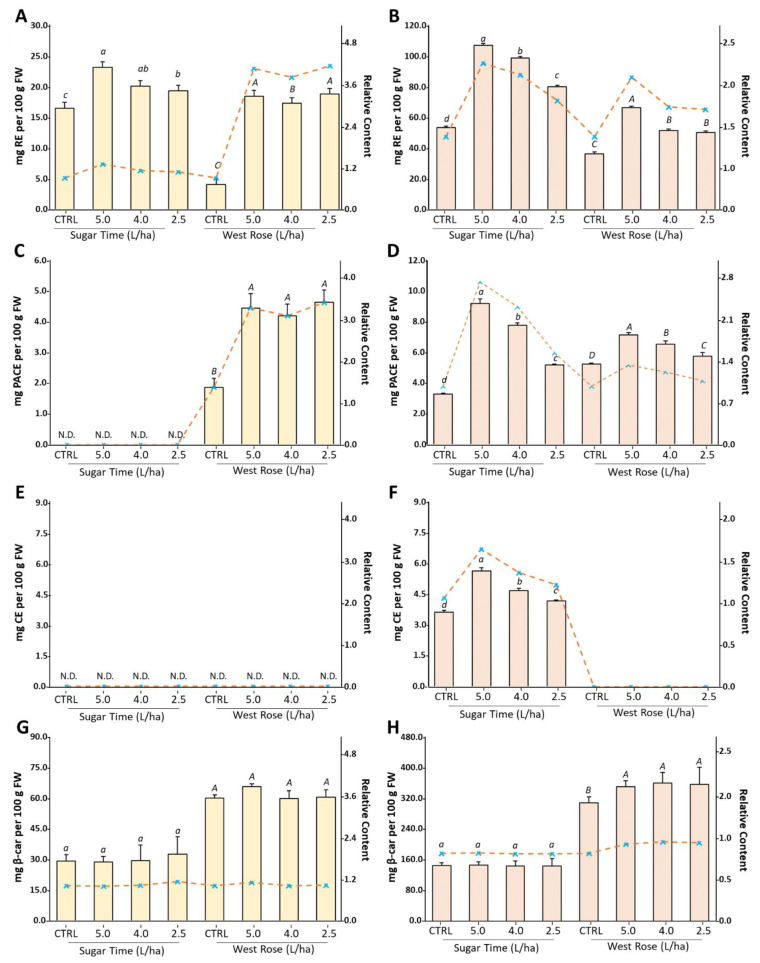
Total content of flavonoids (**A**,**B**), flavan-3-ols (**C**,**D**), anthocyanins (**E**,**F**) and carotenoids (**G**,**H**) measured in the pulp (**A**,**C**,**E**,**F**) or peel (**B**,**D**,**F**,**H**) of peaches harvested from trees treated with different dosages of biostimulant (5.0, 4.0 or 2.5 L/ha) or with water alone. In the different panels, CTRL identifies samples harvested from trees treated with water only. Values are represented as the mean ± SD. For each panel, within the same variety (“Sugar Time” or “West Rose”) the different lowercase (var. SugarTime) or uppercase (var. West Rose) letters indicate significant differences at *p* ≤ 0.05, as measured by Tukey’s multiple interval test. The letter “a” indicates the highest value.

The TPC value measured using the Folin–Ciocâlteu assay is effectively influenced not only by the total flavonoid content but also by all compounds having hydroxyl groups linked to an aromatic ring [54]. In order to understand whether the observed changes in the TPC could be related to variations dependent from specific bioactive compounds, the contents of flavonoids (TFC), flavan-3-ols (TPAC), anthocyanins (TAC) or carotenoids (TCC) were spectrophotometrically evaluated on the extracts of both the pulp and peel of the two peach varieties (Figure 3). After treatment with the biostimulant, an increase in the TFC was observed in both the edible tissues and varieties for all of the tested dosages (Figure 3A,B). However, a greater effect was remarked in the “West Rose” variety. Specifically, the TFC increased by approximately1.2-fold in “Sugar Time” pulp after treatment with the biostimulant and more than three-fold in the “West Rose” pulp (Figure 3A). Similar results were also measured in the peel, in which the boost in the TFC was statistically (*p* < 0.05) more evident. In this case, increments of approximately 2-fold and 1.7-fold were measured for the “Sugar Time” and “West Rose” peels, respectively (Figure 3B). Finally, the observed trend suggests a dose-dependent effect. These results are in accordance with some data reported in the literature.

For instance, Rahman et al. demonstrated that the application of probiotic bacteria as a biostimulant significantly increased the TPC and TFC in treated strawberries [55]. Even Garcia-Seco et al. reported that the application of a microbial biostimulant enhanced the flavonoid metabolism in blackberries [56]. Instead, Nagy et al., investigating the effect derived from the application of four different algal products on apple trees, highlighted that only one of the tested formulations was able to positively affect the TFC [57].

Regarding the content of flavan-3-ols, the DMAC assay revealed that these compounds were exclusively present in the pulp of “West Rose” and in the peel of both varieties (LOD < 0.05 µg/mL; LOQ < 0.14 µg/mL) (Figure 3C,D). The presence of proanthocyanidins in the flesh of a few peach varieties is already well known in literature, and our data are in agreement with previous reports [58]. Similar to the TFC, the TPAC was also positively upregulated by the application of the biostimulant. In particular, the “West Rose” pulp showed a strong increase (approximately 3.2-fold) compared to the CTRL, while a lower rise was observed in the skins. In this case, although the TPAC value of both varieties increased, the most noticeable boost was recorded in the “Sugar Time” peels treated with the highest concentration of biostimulant (Figure 3D). Comparing the effect recorded by biostimulant application on the TPAC in fruits treated with similar formulations appears to be difficult. Indeed, there are no works currently in the literature that have described a similar effect.

Anthocyanins, color pigments found in many edible fruits, are limitedly distributed compounds in the plant kingdom [59]. While not all peach varieties contain anthocyanins, when these pigments are present in the fruits, they are generally localized in the fruit skin in order to protect the most delicate tissues (i.e., pulp and seed) from light and oxidative stresses [59,60,61]. Among the two peach varieties, only the “Sugar Time” fruits had red skins, suggesting the presence of these bioactive compounds only in this plant tissue. This assumption was confirmed by evaluating the TAC via the pH differential method (LOD < 0.03 µg/mL; LOQ < 0.12 µg/mL) (Figure 3E,F). Regarding the treatments with different biostimulant dosages, the TAC was found to be significantly (*p* < 0.05) affected by the application of the formulation (Figure 3F). Indeed, a dose-dependent effect was observed in the peel of the “Sugar Time” fruits, which had a TAC value that almost doubled when the highest dosage was used in the treatment (Figure 3F). In the literature, there are many examples of positive effects of biostimulant treatments on anthocyanidin content. For instance, Frioni et al. tested a biostimulant based on the algae extract and found higher levels of phenolics and anthocyanidins in the skin of “Pinot Noir” and “Cabernet Franc” grapevine cultivars [62]. Regarding other types of biostimulants, Todeschini et al. demonstrated that treatments with a microorganism-based biostimulant could increase the anthocyanidin content in strawberries, in particular by increasing pelargonidin 3-glucoside [63].

Regarding carotenoids (Figure 3G,H), although a positive but nonstatistically significant trend was visible for the “West Rose” peel, our analyses showed that the biostimulant was not able to determine significant (*p* > 0.05) changes in the TCC of both cultivars and for both edible tissues. These data are in accordance with our previous work in which the effects of Expando^®^ was tested on the cv. “Micro-Tom” tomatoes grown in a greenhouse [31]. In addition, in this case we did not find any significant change in the TCC. However, spectrophotometric assays cannot provide quantification of specific forms of carotenoids. Consequently, a change in the carotenoid composition due to the treatment cannot be excluded. Further chromatographic analysis would be necessary to elucidate this point.

### 2.3. HPLC-DAD-MS/MS Analysis Identified Bioactive Compounds Responsible for the Nutraceutical Boost

The potential beneficial effects of phytochemicals on human health that have emerged in recent years have increased both the attention of modern consumers towards plant foods and the interest in their identification and quantification [3,64]. Actually, the study related to the distribution of phytochemical compounds in the plant kingdom appears to be crucial for the valorization of the nutraceutical properties of fruits and vegetables, especially for the species and varieties that are still little known [58,59,65].

**Figure 4:**
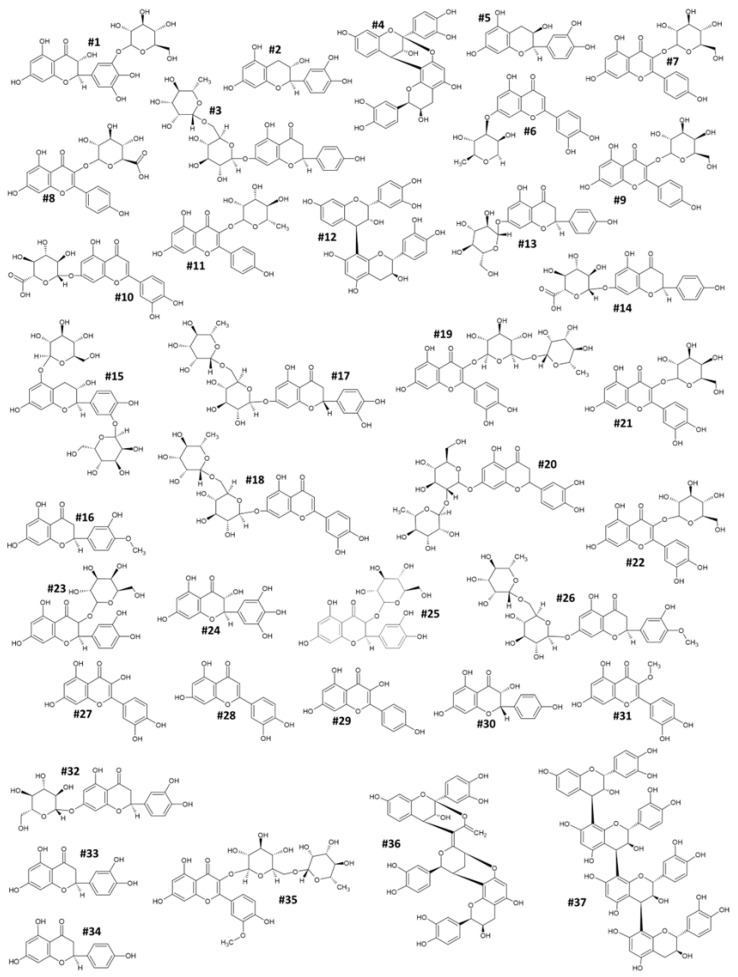
Structure formulae of the bioactive compounds identified and quantified in pulp and peel of peach fruit extracts. The number shown next to the different chemical structures identifies the molecule according to the numbering shown in Table 1.

In this work, in order to identify the phytochemicals contained in the pulp and peel of the two peach varieties (“Sugar Time” and “West Rose”), the same hydroalcoholic extracts of the fruits used for the previous estimation were analyzed by HPLC-DAD-ESI-MS. A chromatographic analysis allowed the identification of 37 different bioactive compounds belonging to various phytochemical classes (Table 1 and Figure 4).

Specifically, nine were flavonols (kaempferol-3-O-glucoside (#7), kaempferol-3-O-glucuronide (#8), kaempferol-3-O-galactoside (#9), kaempferol-3-O-rhamnoside (#11), quercetin-3-O-rutinoside (#19), quercetin-3-O-galactoside (#21), quercetin-3-O-glucoside (#22), quercetin (#27) and kaempferol (#29)); eight were flavanones (naringenin-7-O-rutinoside (#3), naringenin-7-O-glucoside (#13), naringenin-7-O-glucuronide (#14), eriodictyol 7-O-rutinoside (#17), eriodictyol 7-O-neohesperidoside (#20), eriodictyol 7-O-glucoside (#32), eriodictyol (#33) and naringenin (#34)); five belonged to the flavanonol family (Dihydromyricetin-3-O-glucoside (#1), dihydroquercetin-3-O-galactoside (#23), dihydromyricetin (#24), dihydroquercetin-3-O-glucoside (#25) and dihydrokaempferol (#30)); four to flavonones (luteolin-7-O-rhamnoside (#6), luteolin-7-O-glucuronide (#10), luteolin-7-O-rutinoside (#18) and luteolin (#28)), four were proanthocyanidins (PACs) (PAC-A dimer type (#4), PAC-B dimer type (#12), PAC-A trimer type (#36) and PAC-B type tetramer (#37)), three were flavan-3-ols (catechin (#2), epicatechin (#5) and Catechin 3′,5-diglucoside (#15)); two were 4′-methoxy-flavanones ((esperetin (#16) and esperetin 7-O-rutinoside (#26)); one was 3′-methyl-flavonol (3′-O-methylquercetin (#31)); and one was O-methyl-flavonol (isorhamnetin-3-O-rutinoside (#35)) (Table 1, Figure 4). Almost all of the 37 compounds were detected in both cultivars, except for #18, #20, #24, #33 and #37, which were exclusively identified in “Sugar Time” (LOD < 13.35 ng/mL), and #6 in “West Rose” (LOD < 3.14 ng/mL). By analyzing their distribution in the two different tissues and within the same variety, the chromatographic analysis has suggested a differential distribution of bioactive compounds between the pulp and peel. In particular, #22 was identified in the pulp of “Sugar Time”, but it was not present (LOD < 6.23 ng/mL) in the peel. In addition, #7, #24, #28, #30, #33 and #37 were not detected (LOD < 3.21 ng/mL) in the flesh. Concerning “West Rose”, four compounds (#3, #7, #22 and #30) were exclusively identified in the pulp, while seven (#14, #15, #31, #32, #34, #35 and #36) were in the peel (Table 2 and Table 3).

Studies previously published detected some of the identified polyphenolic compounds in the different peach varieties, including “Sugar Time” and “West Rose” [66,67,68]. However, our analysis suggests higher levels of diversity within the different phytochemical classes. For example, the main flavonoids that were before identified in peaches were flavan-3-ols, proanthocyanidins, flavonols and, in some varieties, also anthocyanidins [66,67,68]. Specifically, Tomás-Berberán et al. (2001) detected #2, #5 and #12 in both the pulp and peel, while most flavonols (#21, #22 and #19) were only found in the peel [66]. Similarly, Di Vaio and colleagues (2015) reported the presence of #12 and #2 in peach fruits, along with three quercetin derivatives (#21, #22 and #19) [67]. Furthermore, both studies reported #12 as the most abundant bioactive compound in the peel. The data previously described in the literature are partially in agreement with the quantifications reported in Table 2 and Table 3. In particular, although #12 agrees in being one of the most abundant compounds in the pulp and peel of both peach varieties, #9 and #25 were present in equal amounts in the pulp of “Sugar Time”. Moreover, #7, #21, #23, #25 and #26, along with #12, were also among the major contributors to the total polyphenol content quantified in the peel of this variety (Table 2). Similarly, in “West Rose” other polyphenols have also been quantified in significant amounts, such as #6, #9 and #11 in the pulp, and #1, #6, #8, #10 and #22 in the peel (Table 3).

**Figure 5:**
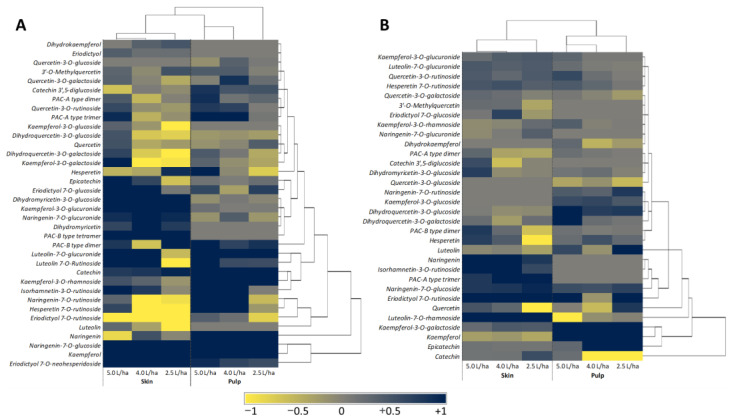
Heat map coupled to a heriodictal analysis showing the relative content of phytochemical compounds identified and quantified in the peel and pulp of peaches harvested from *Prunus persica* trees var. “Sugar Time” (**A**) or “West Rose” (**B**) treated with different concentrations (2.5, 4.0 or 5.0 L/ha) of biostimulant. The relative content was calculated as ln(treated samples/water-only samples), using the values reported in Table 2 and Table 3. The different colors refer to the increase (blue) or decrease (yellow) in the respective molecule. The dendrogram represents the linkage clustering among the treatments (top) or phytochemical compounds (right) using Euclidean distance measures.

The treatment with the biostimulant did not induce qualitative changes in the phytochemical profile of the tested varieties. However, substantial differences in the concentration of specific compounds were recorded (Figure 5). This result should not be surprising. In fact, several papers in the literature agree, highlighting a potential boost at the quantitative level but not in the qualitative profile [59,69,70,71].

On the other hand, it is unlikely that the exogenous application of pure compounds or mixtures of them would lead to extremely profound changes capable of achieving a shift in the secondary plant metabolism to the formation of bioactive molecules normally not present.

From a quantitative point of view, a dose-dependent trend was observed, with the strongest effect in the peaches treated with the highest concentration of biostimulant (Figure 5A).

At this concentration (5.0 L/ha), almost all compounds were increased in both pulp and peel in comparison to the peaches that received only water as a treatment (CTRL). The sole compounds that showed a negative trend in the “Sugar Time” variety were #15, #16, #17 and #34 in the peel and #22, #25, #27, #1 and #14 in the pulp. Regarding “Sugar Time” peel, although the lower biostimulant concentrations (4.0 L/ha and 2.5 L/ha) showed an increase for compounds #1, #8, #14, #37, #2, #13, #29, and #20 quite comparable to that reported for the fruits that were treated with 5.0 L/ha, they had lower values for #7, #25, #27, #23, #9, #10, and #18. More interesting results were obtained for “Sugar Time” pulp. In this case, although the best effects are always most visible in peaches treated with 5.0 L/ha and 4.0 L/ha, also the 2.5 L/ha concentration appeared to be able to increase the content of specific polyphenols without causing the significant reduction of other bioactive compounds, with the exception of #13, #16, #25, and #26. Similar results were also obtained by analyzing the pulp and peel of the “West Rose” variety in the absence of treatment and after biostimulant application (Figure 5B). However, in “West Rose” variety a minor effect on fruit quality was observed. In detail, similar to “Sugar Time” fruits, the most effective treatments on the pulp were the 5.0 and 4.0 L/ha, which allowed an increase of #8, #10, #19, #26, #34, #35, #36, #13, #17 and #6. Although the 2.5 L/ha treatment also resulted in the increase in the same polyphenols, the content of a major number of compounds (#31, #32, #4, #12, #16, #28, #27 and #29) were negatively regulated. Regarding the peel, the chemical analysis revealed that all three biostimulant concentrations were able to equally affect the amount of polyphenols. The only difference linked to the three treatments was related to the content of #2, #21, #22 and #30, which were drastically reduced in peaches that were harvested from trees treated with the lowest concentration (2.5 L/ha) of biostimulant.

### 2.4. The Antioxidant Properties Were Enhanced by the Biostimulant Treatment

Antioxidant activity denotes the ability to maintain the cell structure by fighting free radicals, inhibiting lipid peroxidation and preventing other damages due to the fact of oxidation [72]. In order to evaluate if the application of the biostimulant could increase the content of phytochemicals able to scavenge or reduce free radicals, the antioxidant properties of the edible portions were evaluated via DPPH, ABTS or FRAP assays. Moreover, a cell-based assay was used for quantifying the antioxidant activity of the extracts considering a more physiological system influenced by cellular uptake, distribution and efficiency of protection against peroxyl radicals [3].

**Figure 6:**
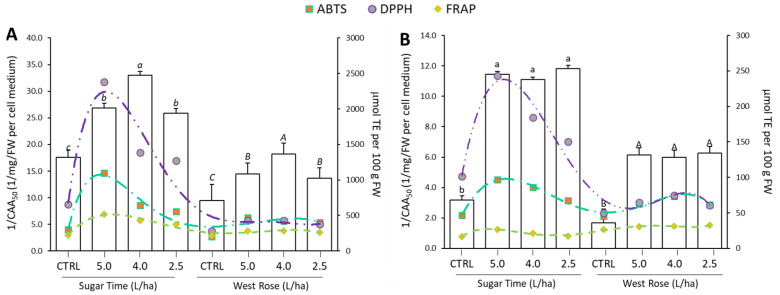
Antioxidant activities of ethanolic extracts from the pulp (**A**) and peel (**B**) from peaches harvested from *Prunus persica* trees var. “Sugar Time” and “West Rose” after treatment with different concentrations (2.5, 4.0 or 5.0 L/ha) of biostimulant or with water alone. In the different panels, CTRL identifies samples harvested from trees treated with water only. The histograms show the cellular antioxidant activity (CAA) expressed as 1/CAA_50_, while the dashed lines indicate the radical scavenging activity (dark green: ABTS; purple: DPPH) or reducing metal (light green: FRAP) measured as µmol TE per 100 g of FW. Within the same series, different lowercase (var. Sugar Time) or uppercase (var. West Rose) letters indicate significant difference at the *p* ≤ 0.05 level as measured by one-way ANOVA followed by Tukey’s multiple range test.

Biostimulant treatments significantly (*p* ≤ 0.05) increased the antioxidant activity, both in terms of radical scavenging and reducing activity (Figure 6). In particular, the DPPH values in the treated “Sugar Time” peaches were 1.89 and 2.56 times higher compared to the CTRL, respectively, in the pulp (Figure 6A) and peel (Figure 6B). Similar results were also observed for the ABTS and FRAP assays (Figure 6).

With regard to the different treatments, a dose-dependent effect was observed for both the pulp and peel extracts of the “Sugar Time” peaches, while for the “West Rose” peaches a boost not dependent on the used dosage was recorded. Comparing the boost recorded for the pulp and peel within the same variety, the higher increase was always found in the treated skins. A number of papers demonstrated that biostimulants are able to improve the antioxidant capacity in plants [73]. For instance, Tarantino et al. found that treatments with three different commercial biostimulants significantly improved the antioxidant properties of apricot fruits (*Prunus armeniaca* L., cultivar “Orange rubis^®^”). In particular, they tested two polysaccharides-based products, one based on carboxylic acids and the other one based on humic acids [43]. In another study, Graziani et al. showed that treatments with an algae-based biostimulant significantly increased the antioxidant capacity of Annurca apple skins. However, they did not find an increase in the pulp. Instead, with a protein hydrolysate-based product, they found a significant increase in the antioxidant activity both in the pulp and in the skin [52]. Other studies demonstrated the positive effects of microbial biostimulants on fruit antioxidant capacities [53]. Rahman et al. tested the effects of two probiotic bacteria on strawberry plants and found that inoculations of the microbial biostimulants increased the scavenging free radical capacity of the fruits [55]. Regarding the same biostimulant investigated in our study, Mannino et al. found an increase in the free radical scavenging activity measured by DPPH and ABTS assays but did not register a significant increase in the reducing activity. In fact, the FRAP values of the treated tomatoes were similar to the untreated ones. Authors affirmed that, probably, this result could be related to the different phytochemicals that were boosted following the application of the biostimulant [31]. Cell-based assays, such as the cellular antioxidant activity (CAA) assay, are gaining increasing relevance, as they provide a more physiological perspective of the oxidative status. The CAA assay is an extremely attractive assay method that can support antioxidant research before using animal models or human clinical trials, because it shows high physiological quality in measuring the antioxidant activity [74,75]. Consequently, this assay is extremely useful, especially in testing extracts from natural products, foods and dietary supplements [3,76,77,78]. In this work, the CAA assay was employed with the aim to assess whether the boost of secondary metabolism observed in the edible tissues of peaches after the application of the biostimulant could be able to increase the number of phytochemicals capable of crossing the cell membrane and counteract the oxidative stress generated by the addition of ABAP. Our analyses revealed that an increase in the 1/CAA_50_ value was recorded for all extracts obtained from both peach edible tissues. This effect was recorded for both the “Sugar Time” and “West Rose” varieties (Figure 6). Similar to the in vitro antioxidant assays, the greatest boost was measured for fruit peel (Figure 6B), which recorded a six-fold and three-fold increase compared with the CTRL for “Sugar Time” and “West Rose”, respectively. On the other hand, although the pulp extracts of both varieties showed an increase in the 1/CAA_50_ value over the CTRL, this increase was smaller (ranging from 1.5-fold to 2.0-fold). Finally, a dose-dependent effect was not recorded for both varieties.

### 2.5. PCA Revealed a Statistical Change in the Nutraceutical Profile of the Fruits

**Figure 7:**
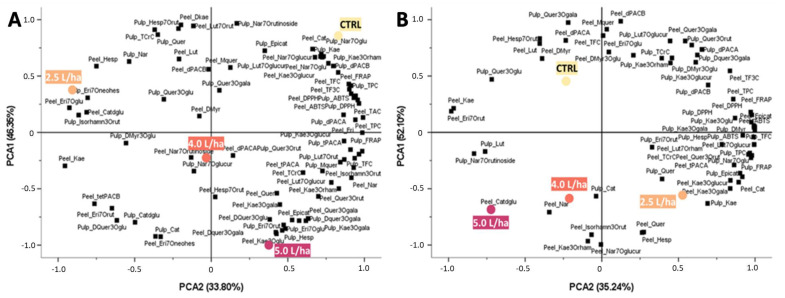
Scatter plot of the principal component (PC1 and PC2) factor scores calculated on the PCA of the phytochemical compounds of the peaches harvested from *Prunus persica* trees var. “Sugar Time” (**A**) or “West Rose” (**B**) treated with different biostimulant concentrations (5.0, 4.0 or 2.5 L/ha) or with water alone. In the different panels, CTRL identifies samples harvested from trees treated with water only. The PCAs were obtained using data relative to both the peel and pulp included and listed in Table 2 and Table 3. Black squares report the distribution of the individual compounds identified via HPLC-ESI-MS/MS analysis. Data used for plotting are reported in Supplementary Materials Table S1 (var. “Sugar Time”) and Supplementary Materials Table S2 (var. “West Rose”).

In order to understand whether the effects observed on the nutraceutical profile of the peaches harvested from *Prunus persica* trees treated with the three different concentrations of biostimulant (5.0, 4.0 or 2.5 L/ha) could be statistically different from that measured on the respective control (CTRL: water only), the data obtained from the spectrophotometric assays (Figure 3 and Figure 6) and those from the HPLC-ESI-MS/MS analyses (Table 2 and Table 3) were used to perform a principal component analysis (PCA) (Figure 7). In “Sugar Time” peaches, the PCA explained 46.35% (PCA1 score) and 33.80% (PCA2 score) of the total variance for a total of 80.15% (Figure 7A).

In agreement with the data reported in the previous sections, the effect derived from the application of the biostimulant in the “Sugar Time” peaches was found to be very pronounced, such that the three treatments (5.0, 4.0 and 2.5 L/ha) and the CTRL were distributed in the four different quadrants. In particular, positive PCA2 and negative PCA1 factor scores effectively differentiated the 5.0 L/ha treatment from both the other treatments (4.0 and 2.5 L/ha) and the CTRL (water only). Furthermore, while maintaining a negative PCA1 and PCA2, the 4.0 L/ha treatment was also discriminated from both the 5.0 L/ha, 2.5 L/ha and CTRL. Finally, the positive PCA1 values allowed the discrimination of the 2.5 L/ha and the control from the treatments in which higher concentrations of biostimulant were used. However, unlike the peaches harvested from trees that were watered only, the “Sugar Time” peaches harvested from the trees treated with 2.5 L/ha were characterized by negative PCA2 values. As suggested by the PCA analysis, the distribution of the different treatments and the collocation of the CTRL in the II quadrant (PCA1 score: positive; PCA2 score: positive) is attributable to both the dose-dependent effect derived from the biostimulant application and the higher amounts of bioactive compounds measured in fruits harvested from trees treated with 5.0, 4.0 or 2.5 L/ha. Specifically, #1, #2, #3, #8, #20 and #29 caused the abovementioned distancing. As for the “West Rose” peaches, the PCA explained 52.10% and 35.24% of the total variance for the PCA1 and PCA2 scores, respectively. In this case, the overall percentage amounted to 87.34% (Figure 7B). Unlike the “Sugar Time” varieties, the peaches of the “West Rose” variety showed a lower distribution, including 5.0 and 4.0 L/ha in the III quadrant (PCA1 score: negative; PCA2 score: negative). Specifically, the fruits harvested from the trees treated with the biostimulant were distributed for the negative PCA2 score values independently from the treatment concentration due to the increase in the content of #8, 10, #19, #26, #34, #35, #36, #13, #17, #27, #6 and #5. However, 2.5 L/ha was discriminated from 5.0 and 4.0 L/ha for the reduction in the content of #31, #32, #4, #12, #16, #28, #27, #29 and #2. This resulted in the appropriate discrimination of the fruits harvested from *Prunus persica* trees treated with 2.5 L/ha from other treatments for the positive PCA2 scores and negative PCA1 scores.

## 3. Materials and Methods

### 3.1. Site Location and Description

**Figure 8:**
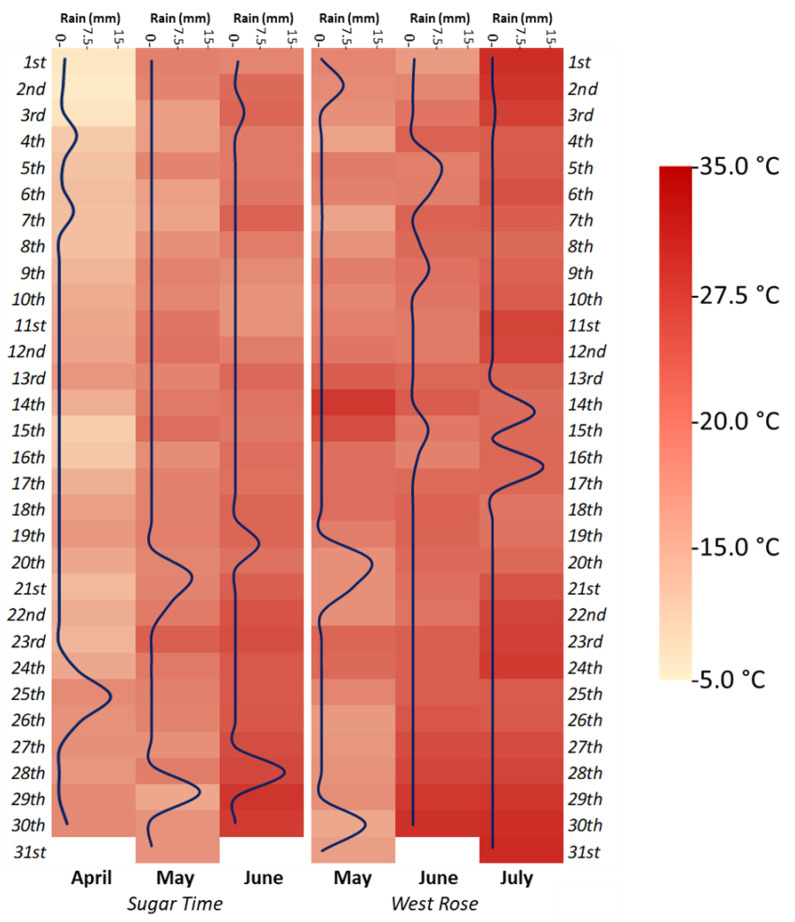
Environmental conditions recorded during the field trial in the two different experimental stations. The different colorings (yellow–red) indicate the different temperatures, expressed as Celsius degrees (°C). In addition, the blue line shows the amount of precipitation (mm per day) that occurred during the experimental months.

The experiments were conducted in 2020 at two different experimental fields located in Italy, Santarcangelo di Romagna (Rimini, Emilia Romagna, Italy) and Canosa di Puglia (Barletta-Andria-Trani, Puglia, Italy). The first experimental site (44°05′64″ N, 12°42′90″ E, 41 m) has a Mediterranean climate, with an average annual temperature of 18.3 °C. The highest monthly temperature is (26–29 °C) in July, while the lowest is (5–8 °C) in January. The annual precipitation is 470.95 mm, with the highest rate in September. The precipitation from April to July accounted for approximately 30% of the total annual precipitation. The average relative humidity was 77%. The climatic conditions during the experimental period are shown in Figure 8. Here, *Prunus persica* trees (“Sugar Time” variety) were planted in 2016 with a planting a rate of 555 P/ha, with a row spacing of 6.0 m. Specifically, the plot size was 90.0 m^2^ (6.0 m × 15.0 m) and composed of 5 plants. The soil texture was clay loam, and its composition was as follows: 35.4% sand, 29.5% silt, 1.80% OM and 33.3% clay. The pH of the soil was 7.95, while the CEC 35.23. The second experimental site (41°08′26″ N, 15°59′37″ E, 146 m) has a temperate climate, with an average annual temperature of 17.4 °C. The highest monthly temperature was (25–31 °C) in August, while the lowest was (4–9 °C) in January. The annual precipitation is 535.69 mm, with the highest rate in December. The precipitation from April to July accounts for approximately 35% of the total annual precipitation. The average relative humidity is 65%. The climatic conditions during the experimental period are shown in Figure 8. Here, *Prunus persica* trees (“West Rose” variety) were planted in 2012 at a rate of 500 P/ha, with a row spacing of 5.0 m. Specifically, the plot size was 60.0 m^2^ (5.0 m × 12.0 m) and composed of 6 plants. The soil texture was clay loam, and its composition was as follows: 37.3% sand, 30.5% silt, 1.79% OM and 32.2% clay. The pH of the soil was 7.45, while the CEC 34.67.

### 3.2. Biostimulant Formulation and Trial Design

The same biostimulant formulation and experimental protocol was used in both trial fields. The treatments were performed with a commercial biostimulant (Expando^®^, GreenHas Group S.P.A., Canale, Italy), already tested in a previous work [31]. The formulation was applied by foliar application using a back-spray sprayer with a volume of water of 1000 L/ha. This application method ensured homogeneous spraying over the leaf surface. The label of the product claims to contain 3% (*w*/*w*) organic nitrogen, 4% (*w*/*w*) phosphoric anhydride, 6% (*w*/*w*) potassium oxide, 0.02% (*w*/*w*) boron, 0.1% (*w*/*w*) molybdenum, 0.02% (*w*/*w*) manganese and 12% (*w*/*w*) organic carbon. The pH (in 1% (*w*/*w*) water solution) and electrical conductivity (in water solution 1 g L^−1^) were, respectively, 6.50 ± 0.50 and 350 µS cm^−1^. The experiments were conducted with four treatments consisting of the application of the biostimulant formulation at different concentrations. Specifically, 2.5 L/ha was applied three times (fruit set, color change and 10 days before harvest), 4.0 L/ha twice (color change and 10 days before harvest) and 5.0 L/ha once (10 days before harvest). Water-only sprayed plants were employed as the controls. All treatments were arranged in a randomized complete block design with four blocks having 30 (6 × 5) unit plots. The distance between two adjacent blocks and plots were 6 and 3 m, respectively. The four treatments were randomly assigned to each plot within the individual blocks with a separate randomization for each block. At the end of the field trial, only fruits considered suitable for consumption were harvested. In order to avoid drift effects, only the middle three plants in each plot were sampled. The fruit numbers, average fruit weight and total yield of each plot was recorded. Immediately after harvest, the fruits were quickly frozen until the extracts for subsequent analysis were prepared.

### 3.3. Preparation of Hydroalcoholic Extracts

In order to perform the extraction of the phytochemical components from the fresh peach fruits, a previously validated procedure that allowed for an exhaustive extraction from a similar plant matrix was used [79]. Briefly, before the extraction process, the peaches were rinsed in deionized water and then slowly thawed at room temperature (RT). The fruits were peeled, and the skin was manually separated from the pulp. Both the peel and pulp were finely chopped and homogenized. For the extraction, 70% (*v*/*v*) EtOH (Sigma-Aldrich, St. Louis, MO, USA) was used as solvent in a 1:10 (*w*/*v*) ratio with the raw plant material. After the addition of the extraction solvent, the samples were vortexed for 30 s, sonicated at RT for 30 min and macerated for 48 h in the dark on an orbiting shaker. The hydroalcoholic extracts were then centrifuged at 5500 rpm for 10 min, and the supernatants were transferred into new clean tubes. The residue obtained from the centrifugation step was extracted twice as described before, and the various supernatants were combined. In order to obtain three biological replicates, the whole extraction process was repeated three times. All extracts were stored at −20 °C until the phytochemical analysis was performed.

### 3.4. Polyphenol Determination

The total polyphenol content (TPC) was determined using the Folin–Ciocâlteu assay [80], suitably modified [58]. In particular, the analytical protocol was optimized for the reading of peach extracts in a multiwell reader (Neo Biotech, Milan, Italy). For the TPC quantification, gallic acid (GA) was used as a reference standard in an external calibration curve (y = 9.99x + 0.04; R^2^: 0.9999; LOD: 19.32·10^−3^ mg/mL; LOQ: 61.87·10^−3^ mg/mL). The TPC content is expressed as mmol GA equivalent (GAE) per 100 g of fresh material (FW) ± standard deviation (SD).

### 3.5. Flavonoid Determination

The total flavonoid content (TFC) was determined by the aluminum chloride method [81]. Here, the original protocol was optimized for the reading of the peach extracts in a microplate reader, as previously described [82]. For the TFC quantification, rutin (Sigma-Aldrich, St. Louis, MI, USA) was used as a reference standard in an external calibration curve (y = 5.30x + 0.05; R^2^: 0.9998; LOD: 15.21·10^−3^ mg/mL; LOQ: 50.19·10^−3^ mg/mL). The TFC content was then expressed as mmol of rutin equivalent (RE) per 100 g of FW ± SD.

### 3.6. Flavan-3-ol Determination

The total flavan-3-ol content (TF3C) was evaluated via a 4-dimethylaminocinnamaldehyde (DMAC) assay [5]. For quantifying TF3C, A2-type Proanthocyanidin (PAC) (Sigma-Aldrich, St. Louis, MO, USA) was used as a reference standard in an external calibration curve (y = 12.57x + 0.07; R^2^: 0.9991; LOD: 5.62·10^−3^ mg/mL; LOQ: 18.55·10^−3^ mg/mL). The TF3C was then expressed as mg of A2-type PAC equivalent (PACE) per 100 g of FW ± SD.

### 3.7. Anthocyanin Determination

The total anthocyanin content (TAC) was assessed using the differential pH method, which allows the spectrophotometric estimation of pigments based on the reversible structural change at different pH environments [59]. Specifically, the original protocol was optimized to quantify the anthocyanins using a multiplate reader, as previously described [27,79], and the obtained absorbances were then used to calculate the TAC value expressed as mg cyanidin equivalent (CYE) per 100 g of FW according to Equation (1):

(1) $$TAC=\frac{(\Delta Abs_{pH=1\;}-\;\Delta Abs_{pH=4.5})\times MW\times DF}{100,000\times \varepsilon \times l\times FW\times 100}$$

where TAC is the total anthocyanin content expressed as mg per 100 g of FW; ΔAbs_pH=1_ is the difference between the absorbance recorded at 510 and 700 nm in KCl buffer; ΔAbs_pH=4.5_ is the difference between the absorbance recorded at 510 and 700 nm in CH_3_COONa buffer; MW is the molecular weight of cyanidin-3-O-glucoside (449.2 g/mol); DF is the dilution factor used before sample injection; 100,000 is the coefficient factor to obtain the desired measurement unit; ε is the molar extinction coefficient factor of cyanidin-3-O-glucoside (26,900 L mol^−1^ cm^−1^); l is the inner distance from the front window to the back window of the spectrophotometric detector (1 cm); FW is the initial fresh weight of the sample.

### 3.8. Carotenoid Determination

For the estimation of the total carotenoid content (TCC), the original homogenized samples of both the peach peel and pulp were extracted with a 50:25:25 *(v*/*v*/*v*) mixture of hexane/acetone/ethanol, using a ratio of 1:10 (*w*/*v*) with the plant raw material, as previously described [1]. Briefly, after vortexing, the samples were centrifuged for 10 min at 5000× *g*, and the upper phase containing the carotenoids was moved into a clean glass tube. The extraction was repeated twice, and the different organic phases were combined together. Consequently, 10 mL of saturated aqueous NaCl (VWR International, Milan Italy) and 5 mL of 10% (*w*/*v*) K_2_CO_3_ (VWR International, Milan Italy) were added to the organic phase. After a quick centrifugation step, the organic phase was dried with an excess of CaCl_2_ (VWR International, Milan Italy). Finally, nitrogen gas was flushed onto the samples in order to remove the excess organic solvent. The whole extraction procedure was repeated twice with the aim of obtaining 3 different technical replicates. Once the samples were prepared, the absorbances at 663, 505 and 453 nm were recorded using a spectrophotometer (Cary 50, Agilent Technologies, Santa Clara, CA, USA). The obtained absorbances were then used to calculate the TCC value expressed as µg carotenoid equivalent (CE) per 100 g of FW according to Equation (2):

(2) $$TCC=0.216\times Abs_{663}-0.304\times Abs_{505}-0.452\times Abs_{453}$$

### 3.9. Identification of Bioactive Compounds via HPLC-ESI-MS/MS

A high-pressure liquid chromatography (HPLC) system was employed for the determination of the phytochemical profile of the peach hydroalcoholic extracts, as previously described [64]. The instrumentation consisted of a liquid chromatography (LC) (Agilent Technologies 1200, Santa Clara, CA, USA) coupled to both a diode array detector (DAD) and an ion trap mass spectrometry (MS) system (Agilent Technologies 6300, Santa Clara, CA, USA) equipped with an electrospray ionization (ESI) source (Agilent Technologies 1200, Santa Clara, CA, USA). The chromatographic separation was carried out using a constant flow rate (0.2 mL min^−1^) through a C18 Luna (Torrance, CA, USA) reversed-phase column (3.00 μm, 150.00 × 3.0 mm i.d.) maintained at 25 °C by a thermostat module (Agilent Technologies 1200, Santa Clara, CA, USA). The UV-VIS spectrum of each compound eluted during the chromatographic run was recorded between 220 and 800 nm. Regarding the mass spectrometry analysis, it was performed operating in the positive mode for the analysis of the anthocyanins and in the negative mode for the analysis of all other polyphenolic compounds. For both types of analysis, the nitrogen flow rate was set at 15.0 mL min^−1^ and the flow temperature was maintained at 350 °C. The capillary voltage was set at ±1.5 kV. The compounds were identified by comparing the retention time (RT), UV-Vis spectra and mass fragmentations of the eluted compounds with those of the authentic reference compounds (Sigma-Aldrich, St. Louis, MO, USA). In addition, their quantification was performed using the calibration curves of the injections of the pure standards. For the analyses of the anthocyanidin compounds, the binary solvent system was Milli-Q H_2_O acidified with 10% (*v*/*v*) formic acid (Sigma-Aldrich, St. Louis, MO, USA) (Solvent A) and 50% (*v*/*v*) MetOH acidified with 10% (*v*/*v*) formic acid (Solvent B). The elution method involved a multistep linear solvent gradient from an initial solvent B concentration of 15% (*v*/*v*) to 45% (*v*/*v*) in 15 min. Finally, the gradient was raised to 70% (*v*/*v*) B in 20 min. The initial solvent concentration was restored at the end of each run and maintained for another 10 min before the next injection. The sample injection volume was 10 μL. For the analysis of the other polyphenolic compounds, the binary solvent system was Milli-Q H_2_O acidified with 0.1% (*v*/*v*) formic acid (solvent A) and acetonitrile acidified with 0.1% (*v*/*v*) formic acid (solvent B). The initial solvent concentration was set to 90% (*v*/*v*) of A for 5 min; then, the solvent concentration was set to 55% (*v*/*v*) in 25 min and finally to 30% (*v*/*v*) in 25 min. The initial solvent concentration was restored at the end of each run and maintained for another 10 min before the next injection. The sample injection volume was 5 μL. The analyses were performed in triplicate.

### 3.10. Evaluation of Antioxidant Properties

The antioxidant properties of the peaches treated with biostimulant were evaluated by measuring the radical scavenging (ABTS and DPPH), reducing metal (FRAP) and cellular antioxidant (CAA) activity of the hydroalcoholic extracts.

#### 3.10.1. 2,2′-Azino-bis(3-ethylbenzothiazoline-6-sulphonic Acid) (ABTS) Assay

The original ABTS assay protocol [83] was optimized for the reading of the samples using a microplate reader, as previously described [26]. Briefly, the ABTS^·+^ reaction mixture was prepared by adding 2.45 M K_2_S_2_O_8_ (VWR International, Milan, Italy) to 7 mM ABTS (VWR International, Milan, Italy) using a 1:2 (*v*/*v*) ratio. After 16 h of incubation in the dark and at RT, the reaction mixture was diluted with EtOH until an absorbance of approximately 0.9 was recorded at 730 nm. Consequently, 100 µL of suitably diluted reaction mixture was incubated with 100 µL of sample. In order to build a dose–response curve, 8 different concentrations were assayed. After 5 min, the 96-well plate was vigorously shaken, and the absorbance at 730 nm was measured. Finally, for each concentration, the color inhibition percentage (CIP) was calculated using Equation (3):

(3) $$\mathrm{CIP}\left(\%\right)=\frac{Abs_{blank}-Abs_{sample}}{Abs_{blank}}\times 100$$

where Abs_blank_ is the absorbance recorded after the injection of 100 µL of pure extraction solvent into 100 µL of reaction mixture; Abs_sample_ is the absorbance recorded after the injection of 100 µL of properly diluted sample into 100 µL of reaction mixture. The obtained percentages were plotted against extract concentrations, and 50% inhibition of the coloration (IC_50_) was calculated via a linear regression analysis. For estimating the ABTS value, Trolox (Sigma-Aldrich, St. Louis, MO, USA) was used as a reference standard in an external calibration curve (y = 596.26x + 20.11; R^2^: 0.9996; LOD: 3.69·10^−3^ mg/mL; LOQ: 10.15·10^−3^ mg/mL). The ABTS value was then expressed as mmol Trolox equivalent (TE) per 100 g of FW ± SD.

#### 3.10.2. 2,2-Diphenyl-1-picrylhydrazyl (DPPH) Assay

The original DPPH assay protocol [83] was optimized for the reading of samples using a microplate reader, as previously described [84]. Briefly, 25 mM DPPH (VWR International, Milan, Italy) reaction mixture was fresh prepared and diluted with EtOH until an absorbance of approximately 1.5 at 520 nm was recorded. Consequently, 100 µL of suitably diluted reaction mixture was incubated with 100 µL of sample. In order to build a dose–response curve, 8 different concentrations were assayed. After 5 min, the 96-well plate was vigorously shaken, and the absorbance at 520 nm was measured. Finally, for each concentration, the color inhibition percentage (CIP) was calculated using Equation (3), and the obtained percentages were used to calculate the 50% inhibition of coloration (IC_50_) via a linear regression analysis. For estimating the DPPH values, Trolox was used as a reference standard in an external calibration curve (y = 221.21x + 7.75; R^2^: 0.9998; LOD: 6.66·10^−3^ mg/mL; LOQ: 18.12·10^−3^ mg/mL). The DPPH value was then expressed as mmol TE per 100 g of FW ± SD.

#### 3.10.3. Ferric Reducing Antioxidant Power (FRAP) Assay

The FRAP reaction mixture was prepared mixing 300 mM sodium acetate (VWR International, Milan, Italy) buffer (pH 3.6) with 10 mM 2,4,6-tris(2-pyridyl)-s-triazine (TPTZ) (VWR International, Milan, Italy) and 20 mM FeCl_3_ (VWR International, Milan, Italy) in a 8:1:1 (*v*/*v*/*v*) ratio [85]. The assay protocol was optimized to monitor the bathochromic shift using a microplate reader, as previously described [3]. Briefly, 170 µL of the FRAP reaction mixture was added to 30 µL of ethanolic extract. After vigorous shaking, the 96-well plate was incubated at 37 °C for 4 min, and the absorbance of each well was recorded at 593 nm. For estimating the FRAP values, Trolox was used as a reference standard in an external calibration curve (y = 7.64x + 0.06; R^2^: 0.9999; LOD: 1.12·10^−3^ mg/mL; LOQ: 3.38·10^−3^ mg/mL). FRAP was then expressed as mmol TE per 100 g of FW ± SD.

#### 3.10.4. Cellular Antioxidant Assay (CAA)

The ethanolic peach extracts were used for the cellular antioxidant activity (CAA) assay, which was performed as previously described [86,87]. Briefly, hepatocellular carcinoma cells (HepG2) were seeded in 96-well plates at a density equal to 6.0·10^4^ cells/well in RPMI medium (Sigma-Aldrich, St. Louis, MO, USA). After 24 h, the medium was removed, and 25 µM 2′-7′dichlorofluorescin diacetate (DCFH-DA) (Sigma-Aldrich, St. Louis, MO, USA) was added in each well along with the different concentrations of the fruit extracts for two hours. In order to remove the potential influence of EtOH, an equal amount of solvent was also added to the culture medium of the wells used as the control or blank. However, in all experimental conditions, EtOH never exceeded 0.25% (*v*/*v*). After the incubation time, the cells were washed twice with phosphate-buffered saline (PBS) (Sigma-Aldrich, St. Louis, MO, USA), and 600 μM 2,2′-azobis(2-amidopropane) (ABAP) (Sigma-Aldrich, St. Louis, MO, USA) dissolved in Hanks’ Balanced Salt Solution (HBSS) was added. The plates were then placed into a plate-reader at 37 °C, and the emission at 538 nm was measured with excitation at 485 nm every 5 min for 1 h. Each plate included a triplicate of the controls and blanks. The control wells were preincubated with 25 µM DCFH-DA and then incubated with 600 μM ABAP in HBSS, whereas the blank wells contained cells treated with 25 µM DCFH-DA in HBSS without the oxidant agent. The area under the curve of fluorescence versus time was integrated to calculate the CAA value at each concentration of the fruit extract using Equation (4):

(4) $$CAA=100-\left[\frac{\int^{\;}SA}{\int^{\;}CA}\right]\times 100$$

where CAA is the cellular antioxidant activity; ∫SA is the integrated area under the curve of fluorescence obtained for samples and normalized for blanks; ∫CA is the integrated area under the curve of the fluorescence obtained for the controls and normalized for the blanks. Finally, the concentration necessary to inhibit 50% of the DCF formation (CAA_50_) for each fruit extract was calculated from the concentration response curves using a linear regression analysis. The data are expressed as CAA_50_ (mg of FW per mL cell medium). The experiments were repeated five times.

### 3.11. Statistical Analysis

Normality and homoscedasticity of the data were assessed by Shapiro–Wilk and Levene’s tests, respectively. ANOVA followed by a Tukey–Kramer’s post hoc HSD test (*p* < 0.05) or Student’s *t*-test was used to determine the significant differences between the samples. To summarize the information obtained from the analytical and biochemical measurement, the PCA was performed using the covariate extraction matrix and varimax rotation. All statistical analyses were performed using the IBM Statistical Package for Social Sciences (SPSS) v. 29.

## 4. Conclusions

In this study, the effect derived from the application of a commercial biostimulant based on seaweed and yeast extracts was investigated on the agronomic performance and nutraceutical profile of fruits harvested from two *Prunus persica* L. varieties grown at field experimental stations. The overall results suggest that the Expando^®^ treatments (i) did not significantly affect the production yield; (ii) achieve a reduction in the ripening time; (iii) induce an increase in the secondary metabolism in both the pulp and peel, resulting in higher nutraceutical and antioxidant properties. The observed effect was evident in the peel compared to the pulp. In conclusion, the application of biostimulant on the two peach varieties proved to be an alternative and sustainable solution for the production of fruits with an increased nutraceutical value that may be more attractive and appealing not only to the modern consumer but also to the food processing industry.

## Figures and Tables

**Table 1:** Phenolic compounds identified and quantified in the pulp and peel of peach fruit samples. The table reports the retention time, mass-to-charge ratio, mass fragmentation pattern, chemical formula, international identification number and the name of the identified molecules. The number listed in the first column refers to the chemical structure of each molecule shown in Figure 4.

No.	RT	m/z	MS/MS	Chemical Formula	CAS-ID	Chemical Name
1	12.6	451	319	C_21_H_22_O_12_	n.a.	Dihydromyricetin-3-O-glucoside
2	13.3	289	261; 245	C_15_H_14_O_6_	7295-85-4	Catechin
3	13.5	595	510.3; 426.4; 342.3; 271.6	C_27_H_32_O_14_	14259-46-2	Naringenin-7-O-rutinoside
4	14.2	575	557; 439; 289	C_30_H_24_O_12_	41743-41-3	PAC-A type dimer
5	14.3	289	261; 245	C_15_H_14_O_6_	490-46-0	Epicatechin
6	15.8	431	395; 329; 293	C_21_H_20_O_10_	n.a.	Luteolin-7-O-rhamnoside
7	15.8	447	400.8; 285	C_21_H_20_O_11_	480-10-4	Kaempferol-3-O-glucoside
8	15.8	461	414.7; 291.7	C_21_H_18_O_12_	22688-78-4	Kaempferol-3-O-glucuronide
9	16.9	447	400.8; 285	C_21_H_20_O_11_	23627-87-4	Kaempferol-3-O-galactoside
10	17.4	461	414.7; 340.7; 298.7	C_21_H_18_O_12_	29741-10-4	Luteolin-7-O-glucuronide
11	18.2	431	384.8; 285	C_21_H_20_O_10_	482-39-3	Kaempferol-3-O-rhamnoside
12	20.2	577	531; 289	C_30_H_26_O_12_	20315-25-7	PAC-B type dimer
13	22.7	403	356.7; 294.7271	C_21_H_22_O_10_	529-55-5	Naringenin-7-O-glucoside
14	23.6	447	404.7; 319; 243.2	C_21_H_20_O_11_	1237479-07-0	Naringenin-7-O-glucuronide
15	24.3	613	571; 553; 289	C_27_H_34_O_16_	105330-54-9	Catechin 3′,5-diglucoside
16	25.0	300	239	C_16_H_14_O_6_	520-33-2	Hesperetin
17	26.8	595	534.8; 287	C_27_H_32_O_15_	13463-28-0	Eriodictyol 7-O-rutinoside
18	27.5	593	344.7; 285.2; 272.6	C_27_H_30_O_15_	20633-84-5	Luteolin 7-O-Rutinoside
19	27.7	609	562.8; 462.7; 300.7	C_27_H_30_O_16_	250249-75-3	Quercetin-3-O-rutinoside
20	27.9	595	463; 449; 287	C_27_H_32_O_15_	13241-32-2	Eriodictyol 7-O-neohesperidoside
21	28.3	463	301.8	C_21_H_20_O_12_	482-36-0	Quercetin-3-O-galactoside
22	28.8	463	301.7	C_21_H_20_O_12_	482-35-9	Quercetin-3-O-glucoside
23	28.8	465	418.8; 396.6; 302.7	C_21_H_22_O_12_	n.a.	Dihydroquercetin-3-O-galactoside
24	29.4	303	285; 257	C_15_H_12_O_8_	27200-12-0	Dihydromyricetin
25	30.4	465	418.8; 396.6; 302.7	C_21_H_22_O_12_	27297-45-6	Dihydroquercetin-3-O-glucoside
26	34.8	609	562.9; 301.4	C_28_H_34_O_15_	520-26-3	Hesperetin 7-O-rutinoside
27	35.0	301	273; 273; 257	C_15_H_10_O_7_	6151-25-3	Quercetin
28	37.6	285	241	C_15_H_10_O_6_	491-70-3	Luteolin
29	37.8	285	269; 257	C_15_H_10_O_6_	520-18-3	Kaempferol
30	37.9	287	269; 231	C_15_H_12_O_6_	104486-98-8	Dihydrokaempferol
31	38.4	315	299; 271	C_16_H_12_O_7_	480-19-3	3′-O-Methylquercetin
32	39.0	449	417; 387; 319: 287.2	C_21_H_22_O_11_	38965-51-4	Eriodictyol 7-O-glucoside
33	39.4	287	269; 241	C_15_H_12_O_6_	552-58-9	Eriodictyol
34	45.4	271	243; 221	C_15_H_12_O_5_	480-41-1	Naringenin
35	46.7	623	554.9; 528.9; 315.4	C_28_H_32_O_16_	604-80-8	Isorhamnetin-3-O-rutinoside
36	54.2	865	575; 557; 439; 289	C_45_H_38_O_18_	37064-30-5	PAC-A type trimer
37	56.3	865	577; 863; 531; 289	C_45_H_38_O_18_	37064-31-6	PAC-B type tetramer

RT: retention time; m/z: charge-to-mass ratio; MS/MS: mass-to-mass fragmentation pattern; CAS-ID: Chemical Abstracts Service Identification number; n.a.: not available.

**Table 2:** Quantitative analysis of polyphenols identified in the fruit peel and pulp of the “Sugar Time” variety harvested from *Prunus persica* trees after treatment with three different concentrations of biostimulant (5.0, 4.0 or 2.5 L/ha) or with water alone. In the different panels, CTRL identifies samples harvested from trees treated with water only. Quantification is expressed as the mean ± SD of three experiments conducted in triplicate, and the values are expressed as mg per 100 g fresh weight (FW). The first column reports the identification number of each compound as listed in Table 1 and shown in Figure 4.

No.	Peel				Pulp			
	CTRL	5.0 L/ha	4.0 L/ha	2.5 L/ha	CTRL	5.0 L/ha	4.0 L/ha	2.5 L/ha
1	3.17 ± 0.19	12.68 ± 0.68	9.49 ± 0.23	12.26 ± 0.28	8.18 ± 0.34	7.05 ± 0.32	8.65 ± 0.47	7.61 ± 0.39
2	1.48 ± 0.07	3.09 ± 0.07	3.25 ± 0.25	3.86 ± 0.08	0.37 ± 0.01	5.98 ± 0.17	2.14 ± 0.07	1.68 ± 0.04
3	7.18 ± 0.08	9.43 ± 0.34	1.32 ± 0.05	2.96 ± 0.15	1.32 ± 0.05	5.61 ± 0.27	4.25 ± 0.29	0.75 ± 0.04
4	4.84 ± 0.29	7.24 ± 0.41	2.87 ± 0.05	4.67 ± 0.22	2.58 ± 0.11	6.16 ± 0.21	2.91 ± 0.18	3.34 ± 0.05
5	2.78 ± 0.14	10.32 ± 0.32	5.1 ± 0.28	1.48 ± 0.05	1.69 ± 0.11	1.91 ± 0.11	1.73 ± 0.05	2.02 ± 0.04
7	58.45 ± 2.76	78.89 ± 3.43	47.01 ± 1.94	20.91 ± 0.58	n.d.	n.d.	n.d.	n.d.
8	3.76 ± 0.23	21.32 ± 0.93	16.29 ± 0.43	12.86 ± 0.81	8.18 ± 0.52	9.44 ± 0.23	8.31 ± 0.25	8.44 ± 0.15
9	41.09 ± 2.63	211.08 ± 12.14	14.73 ± 0.69	16.34 ± 0.89	50.8 ± 3.16	67.14 ± 1.49	43.12 ± 2.85	34.74 ± 1.38
10	14.55 ± 0.54	156.89 ± 8.99	129.21 ± 4.69	8.45 ± 0.29	9.55 ± 0.19	51.55 ± 1.71	60.69 ± 3.52	49.82 ± 1.26
11	80.55 ± 2.39	129.32 ± 8.98	107.14 ± 5.86	65.64 ± 4.56	8.83 ± 0.23	54.37 ± 2.63	56.96 ± 2.62	50.36 ± 2.91
12	224.89 ± 12.28	488.57 ± 13.32	119.13 ± 5.68	1762.13 ± 53.3	48.14 7 1.73	151.86 ± 6.33	91.23 ± 5.52	110.54 ± 7.62
13	3.2 ± 0.21	29.89 ± 1.83	32.46 ± 1.34	16.86 ± 0.91	2.75 ± 0.1	54.33 ± 0.94	26.02 ± 1.16	36.89 ± 0.82
14	3.79 ± 0.17	9.09 ± 0.11	13.01 ± 0.37	9.44 ± 0.21	2.42 ± 0.16	2.01 ± 0.03	3.46 ± 0.21	1.94 ± 0.12
15	4.21 ± 0.15	2.25 ± 0.06	4.32 ± 0.28	3.73 ± 0.25	0.79 ± 0.01	1.77 ± 0.11	1.24 ± 0.04	1.27 ± 0.06
16	5.63 ± 0.36	3.43 ± 0.22	3.89 ± 0.19	13.75 ± 0.56	2.84 ± 0.02	9.53 ± 0.32	2.55 ± 0.17	1.39 ± 0.08
17	29.83 ± 0.64	6.49 ± 0.39	5.2 ± 0.31	6.15 ± 0.35	8.87 ± 0.09	12.38 ± 0.19	10.37 ± 0.44	4.11 ± 0.04
18	2.71 ± 0.16	20.3 ± 0.39	14.01 ± 0.68	0.64 ± 0.01	0.12 ± 0.01	0.89 ± 0.04	0.22 ± 0.01	0.22 ± 0.01
19	28.57 ± 1.94	50.61 ± 2.69	24.79 ± 1.22	22.55 ± 1.37	2.42 ± 0.15	5.08 ± 0.06	4.66 ± 0.32	2.45 ± 0.14
20	0.33 ± 0.01	8.37 ± 0.29	16.95 ± 0.51	2.08 ± 0.02	0.41 ± 0.02	1.02 ± 0.03	0.77 ± 0.01	0.71 ± 0.04
21	58.29 ± 32.48	79.11 ± 30.37	55.51 ± 36.01	38.06 ± 7.83	5.98 ± 0.27	5.87 ± 0.36	13.91 ± 0.79	7.23 ± 0.39
22	n.d.	n.d.	n.d.	n.d.	7.66 ± 0.39	6.47 ± 0.16	10.45 ± 0.68	7.97 ± 0.22
23	85.28 ± 4.47	160.55 ± 6.98	42.31 ± 2.54	23.09 ± 0.3	6.42 ± 0.28	9.51 ± 0.22	6.83 ± 0.42	4.31 ± 0.1
24	0.31 ± 0.01	0.57 ± 0.01	1.45 ± 0.08	0.65 ± 0.01	n.d.	n.d.	n.d.	n.d.
25	82.23 ± 5.28	130.8 ± 2.75	42.19 ± 2.77	38.96 ± 2.58	54.59 ± 1.9	42.93 ± 0.91	44.92 ± 1.31	41.59 ± 2.59
26	30.37 ± 0.69	55.79 ± 1.56	2.63 ± 0.04	4.25 ± 0.14	1.22 ± 0.03	3.37 ± 0.07	4.14 ± 0.26	0.96 ± 0.02
27	7.91 ± 0.39	12.81 ± 0.77	5.34 ± 0.23	6.11 ± 0.12	0.66 ± 0.01	0.55 ± 0.03	0.61 ±	0.94 ± 0.05
28	2.34 ± 0.04	2.97 ± 0.19	1.72 ± 0.09	0.05 ± 0.01	n.d.	n.d.	n.d.	n.d.
29	0.18 ± 0.01	1.54 ± 0.08	2.04 ± 0.05	2.98 ± 0.06	8.94 ± 0.22	55.61 ± 2.88	58.92 ± 1.29	50.52 ± 1.79
30	0.28 ± 0.01	0.29 ± 0.01	0.39 ± 0.01	0.45 ± 0.01	n.d.	n.d.	n.d.	n.d.
31	7.81 ± 0.36	10.24 ± 0.55	6.98 ± 0.34	12.58 ± 0.48	1.53 ± 0.08	2.24 ± 0.13	2.21 ± 0.08	1.52 ± 0.01
32	0.45 ± 0.02	1.62 ± 0.08	1.97 ± 0.05	0.48 ± 0.01	0.51 ± 0.01	0.93 ± 0.04	0.37 ± 0.02	1.02 ± 0.05
33	0.36 ± 0.02	0.51 ± 0.01	0.43 ± 0.01	0.43 ± 0.02	n.d.	n.d.	n.d.	n.d.
34	1.3 ± 0.04	0.56 ± 0.02	2.13 ± 0.12	1.22 ± 0.02	0.16 ± 0.01	8.78 ± 0.42	2.49 ± 0.11	0.71 ± 0.01
35	5.07 ± 0.07	22.66 ± 1.02	10.61 ± 0.51	4.61 ± 0.24	0.39 ± 0.01	2.82 ± 0.15	2.48 ± 0.04	0.39 ± 0.01
36	0.12 ± 0.01	0.29 ± 0.02	0.07 ± 0.01	0.12 ± 0.01	0.04 ± 0.01	0.16 ± 0.01	0.12 ± 0.01	0.04 ± 0.01
37	0.44 ± 0.01	2.72 ± 0.17	3.07 ± 0.14	3.53 ± 0.15	n.d.	n.d.	n.d.	n.d.

n.d. = not detected.

**Table 3:** Quantitative analysis of polyphenols identified in the fruit peel and pulp of the “West Rose” variety harvested from *Prunus persica* trees after treatment with three different concentrations of biostimulant (5.0, 4.0 or 2.5 L/ha) or with water alone. In the different panels, CTRL identifies samples harvested from trees treated with water only. Quantification is expressed as the mean ± SD of three experiments conducted in triplicate, and the values are expressed as mg per 100 g fresh weight (FW). The first column reports the identification number of each compound as listed in Table 1 and shown in Figure 4.

#	Peel				Pulp			
	CTRL	5.0 L/ha	4.0 L/ha	2.5 L/ha	CTRL	5.0 L/ha	4.0 L/ha	2.5 L/ha
1	32.14 ± 1.97	35.44 ± 1.16	38.16 ± 2.36	31.01 ± 0.54	2.41 ± 0.06	4.84 ± 0.12	2.12 ± 0.06	2.26 ± 0.08
2	8.56 ± 0.48	10.12 ± 0.22	1.16 ± 0.06	0.82 ± 0.05	1.08 ± 0.03	1.23 ± 0.05	1.22 ± 0.07	1.84 ± 0.04
3	0.81 ± 0.01	1.25 ± 0.07	1.09 ± 0.06	1.73 ± 0.07	n.d.	n.d.	n.d.	n.d.
4	3.89 ± 0.11	4.56 ± 0.16	4.15 ± 0.16	3.95 ± 0.19	5.51 ± 0.35	7.13 ± 0.44	3.98 ± 0.06	3.81 ± 0.07
5	1.15 ± 0.06	1.45 ± 0.03	10.16 ± 0.63	9.23 ± 0.46	8.84 ± 0.32	9.91 ± 0.63	9.67 ± 0.32	9.69 ± 0.63
6	74.88 ± 3.17	15.31 ± 0.52	64.44 ± 2.55	71.56 ± 4.68	36.46 ± 1.03	107.02 ± 0.21	104.96 ± 4.67	134.49 ± 8.74
7	7.14 ± 0.31	12.34 ± 0.32	13.15 ± 0.68	10.77 ± 0.74	n.d.	n.d.	n.d.	n.d.
8	69.4 ± 2.72	90.35 ± 1.09	72.91 ± 2.71	73.18 ± 2.35	5.71 ± 0.32	6.89 ± 0.41	8.12 ± 0.22	8.59 ± 0.54
9	19.26 ± 0.85	81.67 ± 1.96	61.05 ± 3.47	60.79 ± 1.94	77.76 ± 3.67	95.12 ± 4.96	115.96 ± 1.91	113.25 ± 1.17
10	67.62 ± 2.59	81.36 ± 1.37	69.26 ± 3.57	66.58 ± 4.21	14.41 ± 0.92	21.82 ± 1.27	19.89 ± 0.58	20.97 ± 0.67
11	15.56 ± 0.95	21.92 ± 1.36	15.04 ± 0.51	15.77 ± 1.09	36.46 ± 2.05	32.26 ± 1.02	34.12 ± 0.72	44.64 ± 1.81
12	172.5 ± 97.08	197.55 ± 48.53	177.27 ± 44.56	182.5 ± 35.87	51.17 ± 2.38	109.17 ± 1.47	63.28 ± 2.01	27.48 ± 0.61
13	2.28 ± 0.09	3.89 ± 0.24	3.75 ± 0.11	4.38 ± 0.23	3.18 ± 0.05	6.75 ± 0.43	6.99 ± 0.47	8.16 ± 0.52
14	n.d.	n.d.	n.d.	n.d.	1.74 ± 0.02	1.54 ± 0.02	1.79 ± 0.07	2.44 ± 0.05
15	n.d.	n.d.	n.d.	n.d.	0.84 ± 0.05	1.44 ± 0.07	0.46 ± 0.01	0.79 ± 0.03
16	2.77 ± 0.18	3.32 ± 0.25	4.56 ± 0.27	3.46 ± 0.21	2.61 ± 0.07	5.23 ± 0.08	3.82 ± 0.09	0.99 ± 0.04
17	2.46 ± 0.03	7.74 ± 0.12	1.98 ± 0.06	7.83 ± 0.45	0.63 ± 0.02	2.41 ± 0.08	2.3 ± 0.14	1.94 ± 0.11
19	3.65 ± 0.08	6.53 ± 0.07	4.28 ± 0.04	3.78 ± 0.23	1.05 ± 0.05	1.56 ± 0.13	1.33 ± 0.06	1.56 ± 0.02
21	9.26 ± 0.22	9.86 ± 0.15	8.73 ± 0.12	7.22 ± 0.19	2.26 ± 0.07	2.85 ± 0.16	2.66 ± 0.04	2.19 ± 0.02
22	61.12 ± 1.07	42.35 ± 0.43	51.33 ± 0.61	35.38 ± 0.86	n.d.	n.d.	n.d.	n.d.
23	3.61 ± 0.08	10.13 ± 0.29	4.33 ± 0.28	3.96 ± 0.07	4.61 ± 0.27	5.52 ± 0.32	3.48 ± 0.08	5.43 ± 0.06
25	4.86 ± 0.25	13.39 ± 0.78	9.83 ± 0.47	10.36 ± 0.37	9.04 ± 0.32	9.25 ± 0.53	7.93 ± 0.18	8.14 ± 0.09
26	10.98 ± 0.23	16.21 ± 0.25	14.06 ± 0.21	11.68 ± 0.28	0.72 ± 0.04	1.11 ± 0.06	1.17 ± 0.02	1.01 ± 0.06
27	1.24 ± 0.05	1.21 ± 0.04	0.71 ± 0.04	2.68 ± 0.06	4.42 ± 0.19	10.05 ± 0.36	8.23 ± 0.12	0.82 ± 0.05
28	0.78 ± 0.01	1.39 ± 0.08	0.65 ± 0.04	5.13 ± 0.26	2.98 ± 0.15	2.72 ± 0.15	2.89 ± 0.16	2.15 ± 0.05
29	0.13 ± 0.01	0.58 ± 0.03	0.43 ± 0.02	0.67 ± 0.04	0.76 ± 0.03	0.56 ± 0.03	0.61 ± 0.02	0.51 ± 0.02
30	0.54 ± 0.02	0.68 ± 0.01	0.33 ± 0.01	0.41 ± 0.01	n.d.	n.d.	n.d.	n.d.
31	n.d.	n.d.	n.d.	n.d.	12.77 ± 0.82	15.4 ± 0.19	15.32 ± 0.67	8.92 ± 0.51
32	n.d.	n.d.	n.d.	n.d.	1.81 ± 0.05	1.96 ± 0.02	3.56 ± 0.25	1.44 ± 0.09
34	n.d.	n.d.	n.d.	n.d.	0.33 ± 0.01	1.69 ± 0.11	1.39 ± 0.04	0.72 ± 0.04
35	n.d.	n.d.	n.d.	n.d.	0.48 ± 0.02	2.12 ± 0.05	2.06 ± 0.12	0.86 ± 0.02
36	n.d.	n.d.	n.d.	n.d.	0.04 ± 0.01	0.08 ± 0.01	0.13 ± 0.01	0.11 ± 0.01

n.d. = not detected.

## Data Availability

Not applicable.

## Supplementary Materials

The following supporting information can be downloaded at: https://www.mdpi.com/article/10.3390/ijms232415911/s1.
