# Supplementary material for: Changes in the Phytochemical Profile and Antioxidant Properties of Prunus persica Fruits after the Application of a Commercial Biostimulant Based on Seaweed and Yeast Extract
Source: Int J Mol Sci. 2022 Dec 14;23(24):15911. doi: 10.3390/ijms232415911 (PMC9779733; doi:10.3390/ijms232415911)
Supplement: Supplementary file 1 [file ijms-23-15911-s001.zip › Supplementary Table S1.pdf]

Supplementary Table S1: Covariance matrix of PCA1 and PCA2 components for Sugar Time variety used for plotting Figure 7, Panel A.

	PCA1	PCA2
Pulp_TPC	0,909	0,415
Pulp_TFC	0,937	-0,229
Pulp_TCrC	-0,369	0,911
Pulp_ABTS	0,931	0,307
Pulp_DPPH	0,964	0,257
Pulp_FRAP	0,986	-0,167
Pulp_DMyr3Oglu	-0,556	-0,095
Pulp_Cat	-0,330	-0,925
Pulp_Nar7Orutinoside	0,166	0,968
Pulp_dPACA	0,864	0,156
Pulp_Epicat	0,349	0,725
Pulp_Kae3Oglucur	0,867	-0,076
Pulp_Kae3Ogala	0,574	-0,783
Pulp_Lut7Oglucur	0,713	0,674
Pulp_Kae3Orham	0,722	0,672
Pulp_dPACB	0,829	0,532
Pulp_Nar7Oglu	0,733	0,675
Pulp_Nar7Oglucur	-0,113	-0,344
Pulp_Catdglu	-0,498	-0,797
Pulp_Hesp	0,463	-0,869
Pulp_Eri7Orut	0,472	-0,821
Pulp_Lut7Orut	0,843	-0,264
Pulp_Quer3Orut	0,909	-0,153
Pulp_Eri7Oneohes	-0,807	0,307
Pulp_Quer3Ogala	0,053	0,374
Pulp_Quer3Oglu	-0,309	0,294
Pulp_Dquer3Ogala	0,632	-0,764
Pulp_DQuer3Oglu	-0,617	-0,780
Pulp_Hesp7Orut	-0,214	0,921
Pulp_Quer	-0,349	0,868
Pulp_Kae	0,725	0,667
Pulp_Mquer	0,869	-0,240
Pulp_Eri7Oglu	0,483	-0,853
Pulp_Nar	-0,536	0,629
Pulp_Isorhamn3Orut	-0,866	0,154
Pulp_tPACA	0,965	-0,193
Peel_TPC	0,986	-0,002
Peel_TAC	0,991	0,132
Peel_TFC	0,901	0,383
Peel_TCrC	0,599	-0,356
Peel_TF3C	0,914	0,343
Peel_ABTS	0,956	0,288
Peel_DPPH	0,942	0,324
Peel_FRAP	0,898	0,429
Peel_DMyr3Oglu	0,719	0,694
Peel_Cat	0,661	0,740
