# Supplementary material for: Changes in the Phytochemical Profile and Antioxidant Properties of Prunus persica Fruits after the Application of a Commercial Biostimulant Based on Seaweed and Yeast Extract
Source: Int J Mol Sci. 2022 Dec 14;23(24):15911. doi: 10.3390/ijms232415911 (PMC9779733; doi:10.3390/ijms232415911)
Supplement: Supplementary file 1 [file ijms-23-15911-s001.zip › Supplementary Table S2.pdf]

Supplementary Table S2: Covariance matrix of PCA1 and PCA2 components for West Rose variety used for plotting Figure 7, Panel B.

	**PCA1**	**PCA2**
Pulp_TPC	0.957	-0.195
Pulp_TFC	0.994	-0.107
Pulp_TCrC	0.458	0.66
Pulp_ABTS	0.92	-0.026
Pulp_DPPH	0.869	0.127
Pulp_FRAP	0.917	-0.394
Pulp_DMyr3Oglu	0.448	0.616
Pulp_Cat	-0.037	-0.567
Pulp_Nar7Orutinoside	-0.834	-0.188
Pulp_dPACA	0.653	0.736
Pulp_Epicat	0.902	-0.363
Pulp_Kae3Oglu	0.977	0.091
Pulp_Kae3Oglucur	0.613	0.622
Pulp_Kae3Ogala	0.998	0.024
Pulp_Lut7Oglucur	0.446	0.812
Pulp_Kae3Orham	0.391	0.658
Pulp_dPACB	0.694	0.42
Pulp_Nar7Oglu	0.978	-0.205
Pulp_Hesp	0.783	-0.106
Pulp_Eri7Orut	0.328	-0.132
Pulp_Quer3Orut	0.587	0.775
Pulp_Quer3Ogala	-0.22	0.974
Pulp_Quer3Oglu	-0.718	0.471
Pulp_Dquer3Ogala	0.623	0.687
Pulp_DMyr	0.993	0.044
Pulp_Quer	0.394	-0.413
Pulp_Lut	-0.761	-0.17
Pulp_Kae	0.69	-0.631
Peel_TPC	0.858	0.295
Peel_TFC	0.009	0.859
Peel_TCrC	0.966	-0.183
Peel_TF3C	0.836	0.546
Peel_ABTS	0.999	0.048
Peel_DPPH	0.972	0.18
Peel_FRAP	0.979	0.155
Peel_DMyr3Oglu	0.206	0.685
Peel_Cat	0.893	-0.442
Peel_dPACA	-0.235	0.816
Peel_Epicat	0.993	0.06
Peel_Lut7Orham	0.974	-0.226
Peel_Kae3Oglucur	0.838	-0.498
Peel_Kae3Ogala	0.839	-0.426
Peel_Lut7Oglucur	0.99	-0.041
Peel_Kae3Orham	-0.091	-0.965
Peel_dPACB	0.123	0.986
Peel_Nar7Oglu	0.979	-0.205
